# Hypoxia-Enhanced N110 Glycosylation of Hemagglutinin Promotes H3N2 Influenza Virus Fitness by Modulating Receptor Binding and Immune Evasion

**DOI:** 10.3390/v18050547

**Published:** 2026-05-08

**Authors:** Ting Zhang, Yihui Fang, Jie Liu, Ao Guo, Bin Yuan, Yanan Zhang, Lihua Ding, Qinong Ye

**Affiliations:** 1 National Key Laboratory of Advanced Biotechnology, Academy of Military Medical Sciences, Beijing 100071, China; 2 Department of Pharmacology, School of Basic Medical Sciences, Anhui Medical University, Hefei 230032, China

**Keywords:** H3N2 influenza virus, N-linked glycosylation, hypoxia, B4GAT1-B4GALT1 complex, viral fitness

## Abstract

The hemagglutinin (HA) of influenza A/H3N2 virus evolves rapidly, with glycosylation driving immune evasion. However, how host microenvironmental cues influence this process remains poorly understood. We identified a novel N-linked glycosylation site at position 110 (N110) in contemporary H3N2 viruses (NSS genotype) that enhances viral fitness by increasing receptor-binding signal, HA cleavage, and replication. Remarkably, hypoxia, which mimics the respiratory tract microenvironment, significantly augments N110 glycosylation. Mechanistically, we identified the B4GAT1-B4GALT1 complex as the key mediator of this modification. Hypoxia upregulates their expression and strengthens their interaction with HA. In ferret models, N110-glycosylated viruses exhibit heightened pathogenicity and evade ancestral antibodies. Furthermore, immunization with N110-containing HA confers broad-spectrum protection, whereas reciprocal immunization is ineffective. Our findings reveal hypoxia-driven glycosylation as a previously unrecognized mechanism of H3N2 adaptation, providing critical insights for vaccine efficacy and highlighting the importance of integrating microenvironmental factors into future antiviral strategies.

## 1. Introduction

The H3N2 influenza virus, first emerging in 1968 and causing the Hong Kong flu pandemic, has remained a persistent global pathogen driving seasonal epidemics [1,2,3,4]. Since its emergence, H3N2 has undergone continuous evolutionary adaptation through genetic reassortment and point mutations, with notable divergence in hemagglutinin (HA) glycosylation patterns, a key determinant of immune evasion and host adaptation [5]. Initially conserved, HA glycosylation sites have dynamically shifted over time: existing sites have been lost or modified, while novel sites have emerged, directly shaping viral antigenicity and virulence in response to host immune pressure [6,7].

The functional impact of HA glycosylation is well-documented. For instance, glycosylation at position 158 enhances antibody evasion by masking neutralizing epitopes [8], while modifications at 165 promote antigenic drift by altering receptor-binding specificity [9]. Glycosylation at position 144, acquired during the 1980s–1990s, increased pathogenicity by stabilizing HA structure under acidic conditions, facilitating endosomal fusion and viral entry [10]. Collectively, these modifications enhance viral fitness by balancing receptor binding, immune evasion, and structural stability; such processes complicate vaccine development and diagnostic accuracy because traditional strategies struggle to track rapidly evolving glycoepitopes [11]. Beyond genetic and immune pressures, microenvironmental cues within the host may modulate viral glycosylation. Influenza infection induces pulmonary tissue damage and metabolic stress, creating a hypoxic microenvironment [12,13]. While hypoxia is known to alter host cell glycosylation machinery, its impact on viral protein modifications remains largely unexplored. This knowledge gap is critical, as oxygen-deprived conditions could drive context-specific glycosylation changes that enhance viral replication or immune evasion in vivo.

The 2023–2024 influenza season in China, dominated by H3N2 variants, underscores the urgency of understanding novel glycosylation events. Here, we identify a novel N-linked glycosylation site at position N110 in contemporary H3N2 HA. Functional analyses reveal that N110 glycosylation enhances receptor binding signal, particularly for human 2,6-linked sialic acid receptors, accelerates HA cleavage and cell fusion, and increases viral replication in hypoxic environments. In ferret models, this modification correlates with heightened pathogenicity, including severe weight loss and respiratory symptoms. Importantly, N110 glycosylation alters antigenic presentation, reducing cross-neutralization by antibodies induced by ancestral strains or current vaccines.

Our findings highlight the dual role of N110 glycosylation in viral adaptation: it promotes fitness through enhanced replication and receptor binding while enabling immune evasion via altered epitope exposure. By demonstrating hypoxia-dependent modulation of N110 glycosylation, this study reveals a novel host–virus interaction axis that may drive seasonal H3N2 outbreaks. These insights emphasize the need to integrate microenvironmental factors into glycosylation research and inform next-generation vaccine design targeting evolving glycoepitopes.

## 2. Materials and Methods

### 2.1. HA Sequence Analysis

Approximately 145,000 HA protein sequences of human H3N2 viruses were downloaded from the GISAID database: https://www.gisaid.org (accessed on 17 October 2024). Multiple sequence alignments were generated using MAFFT v7.526, and sequence patterns at positions 110–112 were analyzed using a Perl script. The frequency of each genotype (Y110, N110, N112, and S112) was stratified by year.

### 2.2. Cell Cultures

Lung epithelial cells (A549, CRM-CCL-185), Madin-Darby canine kidney cells (MDCK, CCL-34) and human embryonic kidney cells (HEK293T, CRL-11268) were purchased from American Type Culture Collection (ATCC, Manassas, VA, USA). All cell lines were cultured in complete medium [DMEM containing 10% FBS (FSP500, ExCell, Shanghai, China) and 1% Penicillin-Streptomycin-Amphotericin B Solution (C0052, TargetMol, Boston, MA, USA)] at 37 °C with 5% CO_2_. For experiments under different oxygen tensions, cells were cultured in either normoxic (21% O_2_) or hypoxic (5% O_2_) conditions. Normoxic cultures were maintained in a standard humidified incubator. Hypoxic conditions were established and maintained using a dedicated tri-gas incubator (Heracell™ VIOS 160i, Thermo Scientific, Waltham, MA, USA), which was flushed with a gas mixture of 5% CO_2_, 5% O_2_, and 90% N_2_. The oxygen concentration was continuously monitored and allowed to stabilize for at least 24 h before initiating experiments. All cells were routinely tested for mycoplasma contamination.

### 2.3. Antibodies

Rabbit polyclonal anti-H3N2 HA (11056-T62) and anti-NP (11675-T62) were obtained from Sino Biological (Beijing, China). HRP-conjugated anti-FLAG (A8592) was purchased from Sigma-Aldrich (St. Louis, MO, USA). HRP-conjugated anti-MYC (sc-40HRP) and anti-β-actin (sc-47778HRP) were purchased from Santa Cruz (Santa Cruz, CA, USA). V5-tag Polyclonal antibody was purchased from Proteintech (Rosemont, IL, USA). Rabbit polyclonal anti-B4GAT1 (D263257) was obtained from Sangon Biotech (Shanghai, China). Rabbit polyclonal anti-B4GALT1 (PAB85Hu01) was obtained from Cloud-Clone (Wuhan, China). Mouse monoclonal anti-HIF-1α (610958) was obtained from BD Biosciences (San Jose, CA, USA).

### 2.4. Viruses and Plasmids

Recombinant H3N2 viruses (YSN and NSS) were generated using reverse genetics. The eight viral gene segments (PB2, PB1, PA, HA, NP, NA, M, NS) were amplified from A/Wujiaqu/XJ58/2017 (XJ58) strain by RT-PCR (primers in Table S1) and cloned into the pHW2000 vector, as described by Won-Suk Cho et al. [14]. The HA-NSS variant was constructed via site-directed mutagenesis (Mut Express II Fast Mutagenesis Kit V2, C214-01, Vazyme, Nanjing, China) using YSN HA as the template (primers in Table S2). For rescue, HEK293T cells (95% confluent) were co-transfected with eight pHW2000 plasmids using Lipofectamine 3000 (L3000150, Invitrogen, Carlsbad, CA, USA). At 24 h post-transfection, medium was replaced with serum-free DMEM containing 1 μg/mL TPCK-trypsin (Thermo Scientific, 20233). Supernatants were harvested at 48–72 h and passaged in MDCK cells for amplification. All experiments were performed in a BSL-2+ facility.

B4GAT1 (P15699) and B4GALT1 (P57791) eukaryotic expression vector was purchased from MiaoLing Plasmid Platform (Wuhan, China). Eukaryotic expression plasmids encoding FLAG-tagged, MYC-tagged, or V5-tagged proteins were cloned into pcDNA3.1 (Invitrogen, V79020). FLAG-HA-NSS was generated by mutating FLAG-HA-YSN (Mut Express II Kit, Vazyme C214-01; primers in Table S2). For knockdown experiments, shRNA target sequences are listed in Table S1. The lentiviral shRNA vectors were cloned into the pSIH-H1-Puro (System Biosciences, Palo Alto, CA, USA). For lentiviral infection, HEK293T cells were co-transfected with recombinant lentiviral vector and pPACK Packing Plasmid Mix (System Biosciences) using Megatran reagent (TT210003, Origene, Rockville, MD, USA) to obtain lentiviruses. Viral supernatant was used to infect A549 cells in the presence of polybrene (10 μg/mL). Stable cell lines were obtained following puromycin selection at 1 μg/mL.

### 2.5. Glycosylation Analysis

Proteins were extracted in 8 M urea with 10% protease inhibitor, centrifuged at 14,000× *g* for 20 min, and quantified via Bradford assay. Samples were reduced with 200 mM DTT (37 °C for 1 h), diluted 4 times with 25 mM ammonium bicarbonate, and digested with trypsin (1:50 *w*/*w*; V5111, Promega, Madison, WI, USA) overnight at 37 °C. Digestion was terminated with 50 μL 0.1% formic acid (FA). The resulting peptides were purified using a C18 column (28105-204630, Thermo Scientific), eluted with 70% acetonitrile (ACN), and then subjected to glycopeptide enrichment using a HILIC enrichment column (97502-052130, Thermo Scientific). Eluates were lyophilized, resuspended in 0.2% FA, and analyzed via Liquid Chromatography-Tandem Mass Spectrometry (LC-MS/MS) on a quadrupole Orbitrap mass spectrometer (Thermo Scientific). The mass spectrometer was operated in “top-40” data-dependent mode. Briefly, the Orbitrap mass analyzer (120,000 resolution, 350–1500 *m*/*z* range) collects MS1 (full-scan) spectra with an automatic gain control (AGC) target of 3E6 and a maximum ion injection time of 80 ms. The most intense ions from the full scan were isolated with an isolation width of 1.6 *m*/*z*. Following higher-energy collisional dissociation (HCD) with a normalized collision energy (NCE) of 27, MS2 (fragmentation) spectra were collected in the Orbitrap (15,000 resolution) with an AGC target of 5E4 and a maximum ion injection time of 45 ms. Precursor dynamic exclusion was enabled with a duration of 16 s. Data were searched against the UniProt Homo sapiens database (https://www.uniprot.org/uniprotkb?query=Homo+sapiens, accessed on 21 September 2024) using Byonic40 (1% FDR, Byonic score > 300). Quantitative analysis was performed via R version 4.3.2 using Byonic v4.2.0 and MaxQuant 2.4.0.0 values.

### 2.6. PNGase F Treatment, HA Cleavage Assay, and Immunoblotting

For peptide N-glycosidase F (PNGase F) treatment, virions were denatured in 3 × reducing SDS loading buffer (94 °C for 5 min), cooled, and treated with PNGase F (NEB, P0704S) per manufacturer instructions. Proteins were resolved via SDS-PAGE (Bio-Rad system, Hercules, CA, USA).

For HA cleavage assay, A549 cells infected with recombinant viruses for 24 h were treated with 5 μg/mL TPCK-trypsin for 15 min, lysed in RIPA buffer with protease inhibitors, and analyzed via immunoblotting.

For immunoblotting, proteins were extracted in RIPA buffer, quantified via BCA (Vazyme, E112), and separated via SDS-PAGE. Proteins were transferred to nitrocellulose membranes, blocked in 5% skim milk, and probed with primary/secondary antibodies. Signals were detected using the SuperPico ECL Chemiluminescence Kit (E422-01, Vazyme, Nanjing, China) and imaged using ChemiDoc Imaging Systems with Image Lab 2.0 (Bio-Rad, Hercules, CA, USA).

### 2.7. Plaque Assay

MDCK monolayers in 6-well plates were washed with PBS, inoculated with virus dilutions (serum-free DMEM + 1 μg/mL TPCK-trypsin), and incubated at 37 °C for 1 h (shaking every 15 min). Cells were overlaid with DMEM containing 2% low-melting agarose (Sigma-Aldrich, A9414), 1% FBS, and 1 μg/mL TPCK-trypsin. Plates were incubated at 37 °C for 3 days, fixed with 4% paraformaldehyde (P0099, Beyotime, Shanghai, China), stained with 0.5% crystal violet (Y268090, Beyotime), and plaques were counted by manual counting of the original plates on a high-contrast light box, where plaques were clearly discernible.

### 2.8. Virus Replication Kinetics

A549 and MDCK cells were infected with the recombinant viruses in serum-free DMEM supplemented with 1 μg/mL TPCK-trypsin. After adsorption of viruses for 1 h at 37 °C, cells were washed with PBS buffer and maintained in complete medium at 37 °C for various time points. Then supernatants were harvested to determine the virus titer by performing plaque assay in MDCK cells.

### 2.9. Immunofluorescence

Cells were grown on glass coverslips and fixed in 4% paraformaldehyde fix solution for 15 min at room temperature and permeabilized with Immunostaining Permeabilization Buffer with Triton X-100 (I997471-100 mL, Macklin, Shanghai, China) for 10 min and blocked in sheep serum working solution (ZLI-9056, ZSGB-BIO, Beijing, China) for 30 min. For immunofluorescence staining of NP, cells were incubated in NP antibody and Alexa Fluor 488-conjugated secondary antibodies (A-11094, Invitrogen) diluted in blocking buffer, respectively. For co-localization immunofluorescence staining of B4GAT1 and B4GALT1, cells were incubated in B4GAT1 antibody and Alexa Fluor 488-conjugated secondary antibodies diluted in blocking buffer, respectively. Then, cells were fixed in 2% paraformaldehyde fix solution for 15 min and incubated in 25 mM Glycine solution for 15 min at room temperature. Next, cells were incubated in B4GALT1 antibody and Alexa Fluor 594-conjugated secondary antibodies (A-11012, Invitrogen) diluted in blocking buffer, respectively. The nuclei were stained with 5 μg/mL DAPI solution (R20274, Yuanye Bio, Shanghai, China) for 5 min, before being mounted on glass slides using Antifade Solution (C1210, APPLYGEN, Beijing, China). Imaging was performed using a fluorescence microscope and images were analysed using Image J software (version 2.14.0).

### 2.10. Enzyme-Linked Immunosorbent Assay (ELISA)

Microtiter 96-well plates (260887, Thermo Scientific) were coated with 0.5 μg recombinant HA-YSN/NSS proteins at 4 °C overnight. For HA-sialic acid receptor binding assay, plates were washed with cold wash buffer and incubated with serial dilutions of biotinylated Sialyl-N-acetylactosamine (SLN) (3′-SLN-biotin SCGC-101115, 6′-SLN-biotin SCGC-101198, Scrbio, Shanghai, China) for 1.5 h at 4 °C. After washing, plates were incubated with HRP-streptavidin (35105ES, Yeasen, Shanghai, China) for 1 h at room temperature in the dark. For sera antibody binding assay, plates were washed with cold wash buffer and incubated with serial dilutions of ferret sera for 1.5 h at 4 °C. Subsequently, plates were incubated with HRP-conjugated Goat Anti-Ferret IgG (H+L) (ab112770, Abcam, Cambridge, UK) for 1 h at room temperature in the dark. For both assays, after a final wash, 100 μL of TMB substrate solution (PA107, TIANGEN, Beijing, China) was added to each well, and the plates were incubated at room temperature in the dark for 10–15 min to allow color development. The reaction was terminated by adding 50 μL of 2 M sulfuric acid to each well, and the absorbance was immediately measured at 450 nm using a Multiskan^TM^ FC Microplate Photometer (Thermo Scientific).

### 2.11. Syncytium Assay

A549 and MDCK cells stably expressing copGFP were infected with recombinant viruses. At 24 h post-infection, cells were then washed several times with PBS buffer and treated with 5 μg/mL of TPCK-trypsin for 5 min at 37 °C. The trypsin was then inactivated by washing with PBS buffer containing FBS. To initiate cell fusion, the cells were treated with acidic PBS (pH 5.2, adjusted with citric acid) for 1 min and then incubated in complete medium at 37 °C for 30 min. Fused cells were observed under a fluorescence microscope (Nikon, Tokyo, Japan).

### 2.12. Transfection and Coimmunoprecipitation

HEK293T or A549 cells were transfected with plasmids using Lipofectamine 3000 (L3000150, Thermo Scientific). At 6 h post-transfection, medium was replaced with a fresh medium. Cells were collected 24 h after transfection for further study. For coimmunoprecipitation, cells were infected with RG viruses or transfected with plasmids as indicated and followed by lysing. The protein extracts were then immunoprecipitated with anti-FLAG^®^ M2 Affinity Gel (A2220, Sigma-Aldrich) or indicated antibody according to the manufacturer’s instructions. Precipitated proteins were analyzed via immunoblotting.

### 2.13. Reverse Transcriptase-Polymerase Chain Reaction (RT-PCR)

Total RNA was extracted from cells using TRIzol (R401-01, Vazyme), and 1 μg RNA was reverse-transcribed using HiScript III All-in-one RT SuperMix Perfect (R333-01, Vazyme). The cDNA products were amplified via PCR using gene-specific primers (Table S3). β-actin was used as a reference.

### 2.14. Protein Expression and Purification

HEK293T cells were transfected with FLAG-tagged HA-YSN/NSS plasmids. At 24 h post-transfection, cells were lysed in IP buffer, centrifuged (12,000 rpm, 4 °C), and supernatants were incubated with anti-FLAG^®^ M2 Affinity Gel (A2220, Sigma-Aldrich) for 4 h at 4 °C. Proteins were eluted with 3 × FLAG peptide (F4799, Sigma-Aldrich) and quantified via BCA.

### 2.15. Immunopurification and Mass Spectrometry (MS)

MS was performed to obtain HA-interacting proteins by Beijing Qinglian Biotech, Co., Ltd (Beijing, China). A549 cells expressing FLAG-HA-YSN or NSS were lysed in IP buffer and immunoprecipitated using anti-FLAG^®^ M2 Affinity Gel (A2220, Sigma-Aldrich), followed by elution with 3 × FLAG peptide according to the manufacturer’s instructions. The eluted proteins were added to NuPAGE 4–12% Bis-Tris gel (Invitrogen, Cat: NP0321PK2) to separate protein complexes, silver stained (24600, Pierce, Rockford, Illinois, USA) and subjected to MS sequencing and data analysis. Briefly, the stained protein bands were cut into 1 mm^3^ pieces and destained with 50% ACN in 50 mM ammonium bicarbonate until the gel turned white. Proteins were reduced with 10 mM DTT at 56 °C and alkylated with 55 mM iodoacetamide at room temperature in the dark. Subsequently, trypsin digestion was performed overnight at 37 °C with gentle shaking. Digested peptides were isolated using 1% trifluoroacetic acid in 50% ACN, vacuum-dried, and reconstituted in 0.1% formic acid. Nanoflow LC-MS/MS analysis of tryptic peptides was conducted on a quadrupole Orbitrap mass spectrometer (Q Exactive HF-X, Thermo Scientific) coupled to an EASY nLC 1200 ultra-high-pressure system (Thermo Scientific) via a nano-electrospray ion source. MS/MS data were generated with a data-dependent analysis mode and analyzed using PLGS 2.4 software, and the resulting peak list was searched against the NCBI database with the MASCOT v2.8 search engine. Interacting proteins are listed in Table S4.

### 2.16. Ferret Models

Ferret in vivo animal model experiments were performed under an animal biosafety level 2+ (ABSL2+) condition in isolator cages by Beijing Vital River Laboratory Animal Technology Co., Ltd (Beijing, China).

For virus infection experiment, 3-month-old seronegative female ferrets (*n* = 3 per group) were anesthetized with isoflurane and inoculated intranasally with 10^6^ TCID_50_ YSN/NSS virus (250 μL per nostril). Weight, temperature and clinical signs were monitored daily. At 12 dpi, serum samples were collected for further study. Subsequently, the ferrets were euthanized. For ferret serum collection, after anesthesia, the ferret is secured dorsally in a biosafety cabinet, and fur from the neck to sternum is clipped. The strongest heartbeat point is palpated and disinfected, followed by blood collection using a 1 mL syringe. The whole blood is transferred to a serum separation tube, left at room temperature for ≥30 min, centrifuged at 10,000 r/min for 5 min, and the supernatant serum is aliquoted and stored at −80 °C.

For vaccine challenge experiment, ferrets were immunized intramuscularly with 5 μg of recombinant HA-YSN/NSS formulated with MF59 adjuvant (AVT) and then boosted three weeks later, the control group received PBS formulated with an equal volume of MF59 adjuvant as a negative control. Three weeks post-boost, ferrets were challenged by intranasal inoculation with 10^6^ TCID_50_ of 250 μL YSN/NSS virus (*n* = 3 per group). Nasal swabs were collected at 1, 3, and 5 dpi for viral titration, and lung tissues were harvested at 5 dpi for viral titration, histopathological and immunohistochemistry analysis. For viral titration, sterile swabs were collected and immersed in preservation medium, then centrifuged at 3000× *g* for 10 min at 4 °C to remove impurities. Lung tissues were weighed and homogenized in ice-cold PBS buffer at a concentration of 10% (*w*/*v*), followed by centrifugation under the same conditions. The supernatants were serially diluted ten-fold in serum-free DMEM. Each dilution was inoculated in quadruplicate onto MDCK cells in 96-well plates and incubated at 37 °C with 5% CO_2_ for 72 to 96 h. Cytopathic effect (CPE) was monitored daily. The viral titer of each sample was calculated as the 50% tissue culture infectious dose (TCID_50_) per milliliter using the Reed-Muench method. Lung tissues were fixed in 4% paraformaldehyde fix solution, embedded in paraffin, and sections were stained with hematoxylin and eosin using a Hematoxylin and Eosin Staining Kit (Beyotime, C0105S) or with anti-NP antibody, photographed by a fluorescence microscope. Pathology scores quantifying lung inflammation were assessed blindly by a pathologist based on alveolar damage, interstitial edema, and inflammatory cell infiltration.

### 2.17. Virus Neutralization Assay

Immune sera were collected from ferrets inoculated with recombinant YSN or NSS type virus and heat-inactivated at 56 °C for 30 min to remove complement. Serial two-fold dilutions of sera were prepared in serum-free DMEM medium, and 50 μL of each dilution was mixed with an equal volume of virus (100 TCID_50_/50 μL or 100 PFU/50 μL). The mixtures were incubated at 37 °C for 30 min to allow neutralization. Preliminary experiments were performed to optimize the virus titer and serum dilution range, and the optimized conditions were applied to subsequent viral infectivity assays.

### 2.18. Statistical Analysis

Data are presented as mean ± SD. A two-tailed Student’s *t* test was used for two-group comparisons, and one-way ANOVA or two-way repeated-measures ANOVA with Bonferroni correction was used for multiple comparisons. *p* < 0.05 was considered significant. Analyses were performed using GraphPad Prism 8.4.0.

## 3. Results

### 3.1. Acquisition of a Novel N-Linked Glycosylation Site in the Hemagglutinin of H3N2 Influenza A Viruses

**Figure 1:**
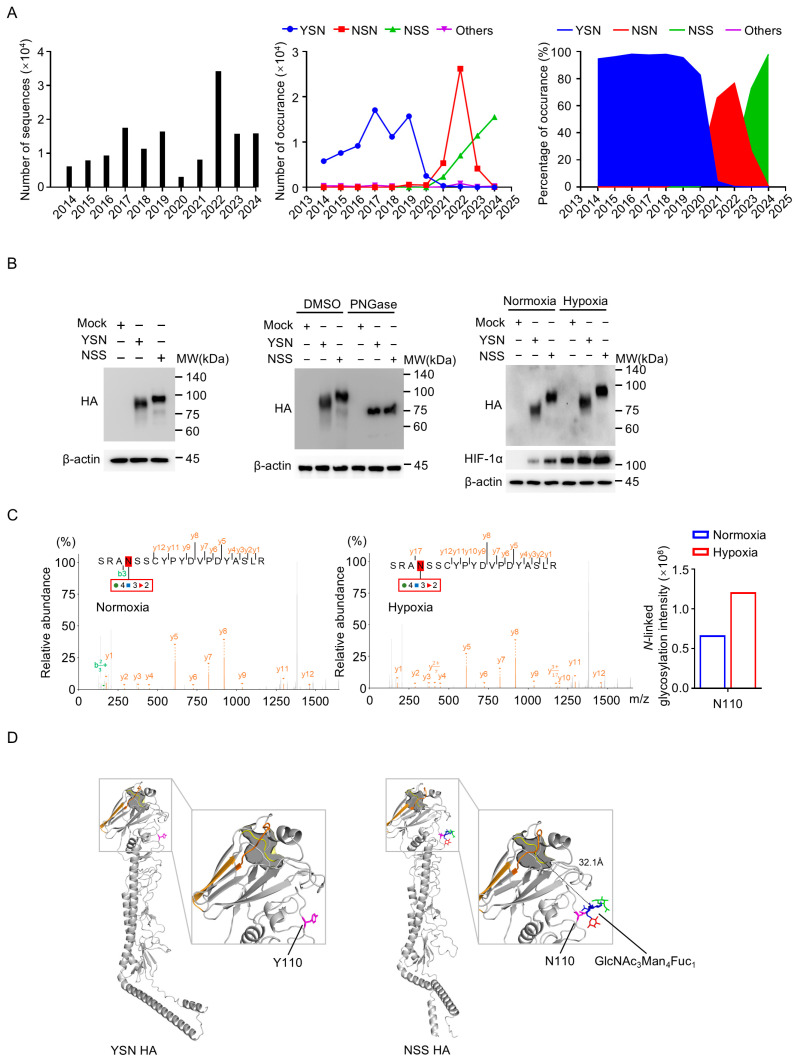
Acquisition of a novel N-linked glycosylation site in H3N2 hemagglutinin. (**A**) Temporal dynamics of HA genotype evolution in H3N2 influenza A viruses. Analysis of ~145,000 HA amino acid sequences from the GISAID database (2014–2024) showing the prevalence of YSN (Y110/N112), NSN (N110/N112), NSS (N110/S112), and other genotypes. (**B**) Immunoblot analysis of HA glycosylation. Electrophoretic mobility of HA proteins from YSN and NSS viruses in A549 cells (**Left** panel). Effect of peptide N-glycosidase F (PNGase F) treatment on HA molecular weight (**Middle** panel). Detection of HA glycosylation and HIF-1α expression under normoxic (21% O_2_) or hypoxic (5% O_2_) conditions (**Right** panel). Cells were infected with YSN or NSS viruses at an MOI of 3, β-actin was used as a loading control. MW, molecular weight. (**C**) Site-specific glycosylation analysis by glycoproteomic mass spectrometry. The red-highlighted “N” indicates the site of glycosylation modification. The glycan compositions are shown at the positions of the modifications, with blue, green, and red representing GlcNAc, Man, and Fuc residues, respectively. Relative abundance of glycans at the HA 110 locus in NSS viruses under normoxia (**Left** panel) and hypoxia (**Middle** panel). The b*_n_* and y*_n_* ions denote fragment ions produced by the cleavage of peptide bonds during MS/MS fragmentation; b*_n_* ions represent fragments where the charge is retained on the N-terminal portion, while y*_n_* ions represent fragments where the charge is retained on the C-terminal portion, with ‘n’ indicating the number of amino acid residues from the respective terminus. (**D**) AlphaFold 3-predicted structures of YSN (unglycosylated) and NSS (glycosylated) HA. A representative 8-saccharide hybrid-type glycan (GlcNAc_3_Man_4_Fuc_1_) was modeled at N110. The glycan is shown in sticks, with blue, green, and red representing GlcNAc, Man, and Fuc residues, respectively. The RBS elements—130-loop (yellow), 190-helix (orange), and 220-loop (bright orange)—are highlighted. The dashed line indicates a 32.1 Å distance between the glycan and RBS.

To investigate the evolution of H3N2 influenza A virus hemagglutinin (HA), we analyzed ~145,000 HA amino acid sequences of human H3N2 isolates from the GISAID database (2014–2024) (Figure 1A). This analysis identified two critical amino acid substitutions at positions 110 and 112: while the YSN (Y110/N112) genotype predominated before 2019, a Y110N substitution emerged in 2020, and reached peak prevalence by 2022. Concurrently, an N112S substitution arose and accumulated annually, leading to the emergence of a double mutant (NSS; Y110N/N112S) that approached near-complete fixation by 2024 (Figure 1A). Notably, the NSS genotype introduces a putative N-linked glycosylation motif (N-X-S/T) at HA position 110, prompting investigations into whether this novel post-translational modification influences viral transmission or immune evasion.

To characterize this glycosylation event, reverse genetics was used to rescue isogenic H3N2 viruses encoding YSN or NSS HA. Immunoblot analysis of infected A549 cells revealed distinct electrophoretic mobility between YSN and NSS HA proteins, with the latter exhibiting a significantly higher molecular weight (Figure 1B, left panel). To confirm N-linked glycosylation, virions were treated with peptide N-glycosidase F (PNGase F), which removes N-linked oligosaccharides. Following PNGase F digestion, the molecular weights of YSN and NSS HA proteins were reduced to comparable levels, confirming the presence of additional N-glycosylation in the NSS variant (Figure 1B, middle panel). Given the hypoxic microenvironment associated with influenza pneumonia, we further evaluated HA glycosylation under low-oxygen conditions (5% O_2_). The results revealed that hypoxia enhanced HA glycosylation in both YSN and NSS viruses, potentially conferring a survival advantage during acute respiratory infection (Figure 1B, right panel). Furthermore, here we observed that the protein accumulation of HIF-1α is significantly higher compared to mock infected cells, a result similar to that observed in H1N1 virus infection, and NSS virus further enhanced this effect. Glycoproteomic mass spectrometry (MS) confirmed site-specific glycosylation at N110 in NSS HA, with no detectable glycans at this residue in YSN HA (Figure 1C). Importantly, hypoxic conditions significantly upregulated glycosylation intensity at N110.

MS analysis confirmed that the N110 site within the NSS HA variant is post-translationally modified by N-linked glycosylation under both normoxic and hypoxic conditions, with a notable increase in glycosylation intensity observed under hypoxia (Figure 1C). To investigate the structural implications of this modification, we utilized AlphaFold 3 to model the HA protein. Based on the MS-derived glycoform composition, we predicted the most likely glycan structure to be a hybrid-type N-glycan (GlcNAc_3_Man_4_Fuc_2_). Due to modeling input constraints, a representative 8-saccharide chain GlcNAc_3_Man_4_Fuc_1_ was mapped onto the N110 residue, localizing it to a solvent-exposed region on the HA globular head (Figure 1D). This specific composition corresponds to a biosynthetic intermediate where the α1–3 arm has been extended by GlcNAc while the α1–6 arm retains three untrimmed mannose residues.

We further examined the spatial relationship between this glycan and the receptor-binding site (RBS), the conserved pocket responsible for recognizing host cell sialic acid receptors [15,16]. Comparative structural analysis revealed that the N110 glycan is positioned on the lateral surface of the globular head, with its terminal atom maintaining a distance of 32.1 Å from the RBS (Figure 1D). This significant spatial separation suggests that the N110 glycan does not cause direct steric hindrance to receptor binding.

### 3.2. Hypoxia-Enhanced N110 Glycosylation Promotes H3N2 Viral Replication and Pathogenicity

**Figure 2:**
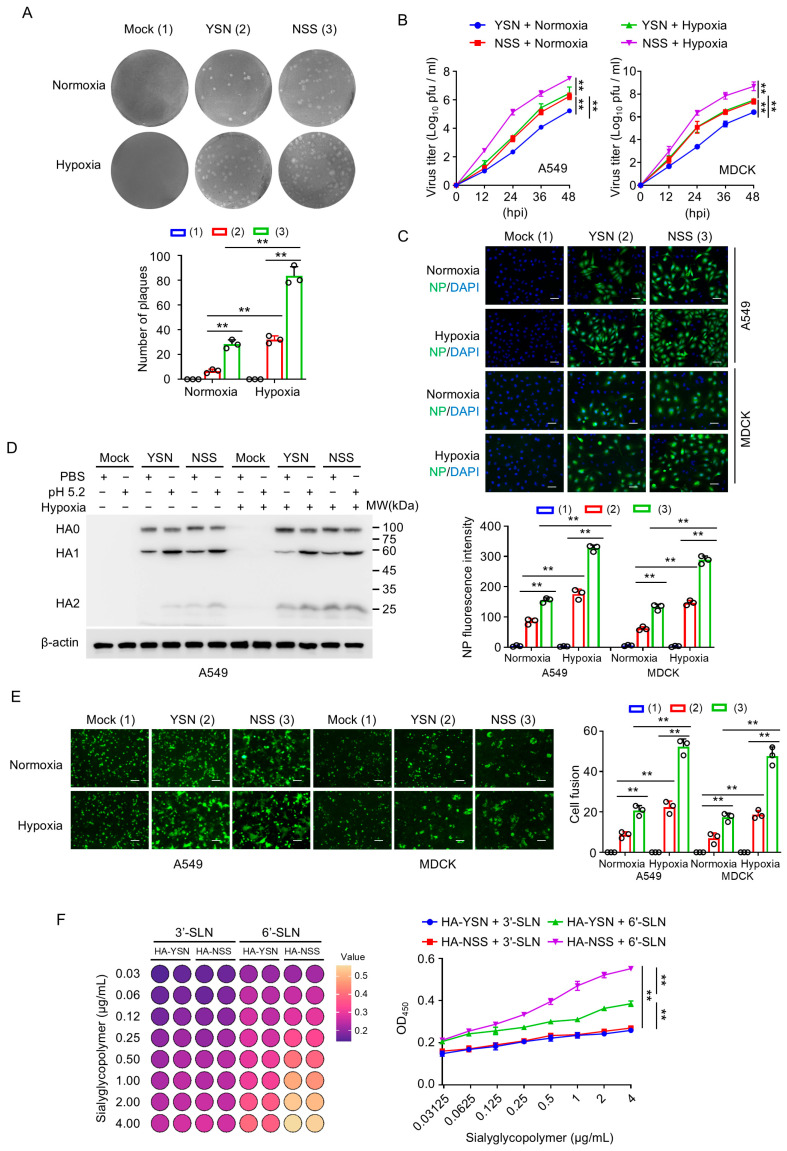
Hypoxia-enhanced N110 glycosylation promotes H3N2 viral replication and pathogenicity. (**A**) Plaque formation assay in MDCK cells infected with YSN or NSS viruses at an MOI of 0.1 under normoxia or hypoxia, and visualized through crystal violet staining. Quantitative analysis of the plaque numbers was carried out (**Bottom** panel), circles indicate values from three replicates. (**B**) Viral replication kinetics in A549 and MDCK cells infected with YSN or NSS viruses at an MOI of 3. Viral titers were measured at 12, 24, 36 and 48 h post-infection (hpi) under normoxia or hypoxia. (**C**) Immunofluorescence staining of viral nucleoprotein (NP). A549 and MDCK cells infected with YSN or NSS viruses at an MOI of 0.1 under normoxia or hypoxia (24 hpi). NP expression (green) was quantified by fluorescence intensity (**Bottom** panel), circles indicate values from three replicates. Nuclei were stained with DAPI (blue). Scale bar, 50 μm. (**D**) A549 cells were infected with YSN or NSS viruses at an MOI of 3 under normoxia or hypoxia. Immunoblot analysis showed the HA cleavage efficiency at pH 5.2 (mimicking endosomal conditions). (**E**) Cell–cell fusion assay in A549 and MDCK cells stably expressing a copGFP reporter. Syncytium formation was induced by pH 5.2 treatment, and the syncytium-forming abilities of viruses were statistically analyzed under normoxic and hypoxic conditions, circles indicate values from three replicates. Scale bar, 100 μm. (**F**) HA binding signal to sialic acid receptors measured by ELISA. The binding of hemagglutinin (HA) to sialic acid receptors was detected by ELISA. The left heatmap visually represents the data in a color-coded format, and the right statistical chart quantitatively illustrates the comparison of binding signal intensities of HA-YSN and HA-NSS to 2,3-linked sialic acid (3′-SLN) and 2,6-linked sialic acid (6′-SLN). Data are presented as mean ± SD of three independent experiments and were analyzed using one-way ANOVA test with Bonferroni correction (**A**,**C**,**E**,**F**) and two-way repeated-measures ANOVA test with Bonferroni correction (**B**). ** *p* < 0.01.

To evaluate the impact of N110 glycosylation on H3N2 viral replication, we compared the growth kinetics of isogenic YSN and NSS viruses in A549 and MDCK cells. Plaque-forming assays revealed that NSS viruses yielded significantly more plaques than YSN viruses (Figure 2A), with enhanced replication efficiency observed at 12, 24, and 48 h post-infection (hpi) in both cell lines (Figure 2B). These data indicate that N110 glycosylation promotes H3N2 viral replication. Notably, hypoxic conditions further increased plaque formation and replication rates in both YSN and NSS viruses, suggesting that low-oxygen microenvironments may exacerbate viral spread during infection (Figure 2A,B). Immunofluorescence staining confirmed these observations, demonstrating higher viral loads in NSS-infected cells compared to YSN-infected cells, with hypoxia further augmenting infection efficiency across both cell lines (Figure 2C).

Since HA cleavage is a critical determinant of viral infectivity, we next assessed whether N110 glycosylation modulates this process. We assessed HA cleavage at pH 5.2, which mimics both the endosomal trigger for fusion and the acidic environment of the upper respiratory tract. At this pH, NSS HA exhibited a trend toward higher cleavage efficiency compared to YSN HA in A549 cells, although the difference was modest (Figure 2D). This finding suggests that N110 glycosylation may enhance viral entry by promoting HA activation. Additionally, hypoxia further increased HA cleavage in both viruses, potentiating viral replication by accelerating this key maturation step (Figure 2D).

In the context of influenza virus, cell–cell fusion refers to the formation of multinucleated syncytia when viral HA expressed on the surface of an infected cell binds to receptors on adjacent host cells, mediating membrane merging that facilitates direct cell-to-cell spread and immune evasion [17,18]. To investigate the effect of N110 glycosylation on viral entry, we analyzed cell–cell fusion mediated by YSN and NSS HA in A549 and MDCK cells. N110 glycosylation significantly enhanced fusion activity, and this effect was further amplified under hypoxic conditions (Figure 2E), indicating that glycosylation-dependent modulation of HA function may facilitate viral spread in oxygen-depleted tissues.

Given the surface localization of N110 (Figure 1D), we examined its impact on HA binding to sialic acid receptors using ELISA. N110 glycosylation (HA-NSS) significantly increased HA binding signal to sialic acid-containing receptors, with a preferential enhancement for 2,6-linked sialic acid (6′-SLN), which was the predominant receptor on human respiratory epithelial cells, compared to 2,3-linked sialic acid (3′-SLN) (Figure 2F). This receptor-binding bias may contribute to the enhanced transmissibility of NSS viruses in humans. In summary, N110 glycosylation enhanced H3N2 viral replication, HA cleavage, cell fusion, and binding to human 2,6-sialic acid receptors, with hypoxic conditions further amplifying these effects, collectively contributing to increased viral fitness and pathogenic potential.

### 3.3. The B4GAT1-B4GALT1 Complex Mediates Hypoxia-Enhanced N110 Glycosylation of H3N2 HA

**Figure 3:**
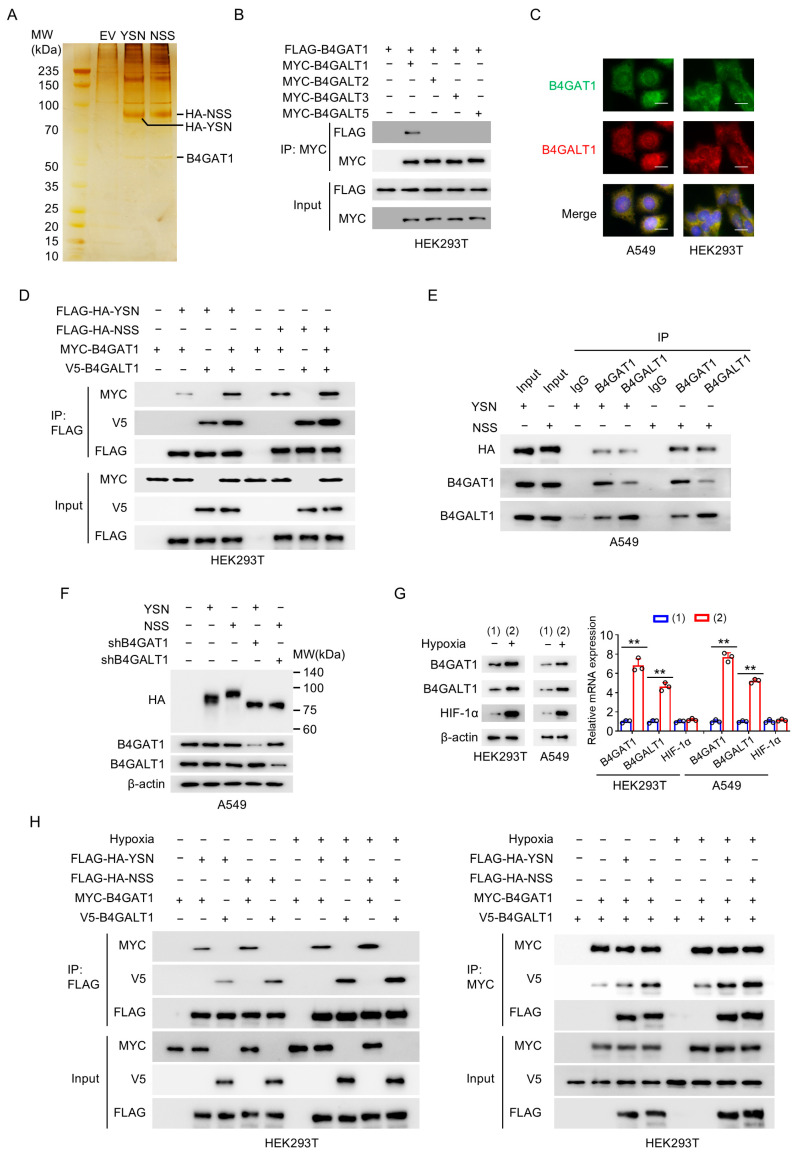
The B4GAT1-B4GALT1 Complex Mediates Hypoxia-Enhanced N110 Glycosylation of H3N2 HA. (**A**) Silver-stained SDS-PAGE gel showing proteins co-immunoprecipitated with FLAG-tagged HA-YSN or HA-NSS from A549 cells. Specific bands were identified by mass spectrometry. EV, empty vector. MW, molecular weight. (**B**) Co-immunoprecipitation (Co-IP) analysis of HEK293T cells transfected with plasmids encoding FLAG-tagged B4GAT1 and MYC-tagged B4GALT family members (B4GALT1-3 and B4GALT5). IP, immunoprecipitation. (**C**) Immunofluorescence staining of B4GAT1 (green) and B4GALT1 (red) in A549 and HEK293T cells. Yellow signals in the merged images indicate colocalization. Nuclei were counterstained with DAPI (blue). Scale bar, 50 μm. (**D**) Co-IP analysis of HEK293T cells transfected with plasmids encoding FLAG-tagged HA (YSN or NSS) and MYC-tagged B4GAT1 and/or untagged B4GALT1. IP, immunoprecipitation. (**E**) Co-IP analysis of endogenous interaction in A549 cells infected with YSN or NSS viruses at an MOI of 3. Co-IP with anti-B4GAT1 or anti-B4GALT1 antibody confirmed the binding of HA-YSN and HA-NSS to endogenous B4GAT1 or B4GALT1. (**F**) Immunoblot analysis of HA glycosylation. Electrophoretic mobility of HA proteins from YSN and NSS viruses in A549 cells stably infected with shRNAs targeting B4GAT1 or B4GALT1. β-actin was used as a loading control. MW, molecular weight. (**G**) Immunoblot (**Left** panel) and RT-qPCR (**Right** panel) analysis of B4GAT1, B4GALT1 and HIF-1α expression levels in HEK293T and A549 cells under normoxia or hypoxia, circles indicate values from three replicates. (**H**) Co-IP analysis of HEK293T cells transfected with plasmids encoding FLAG-tagged HA (YSN or NSS), MYC-tagged B4GAT1 and untagged B4GALT1 as indicated under normoxia or hypoxia. Data are presented as mean ± SD of three independent experiments and were analyzed using Student’s *t* test (**G**). ** *p* < 0.01.

N-linked glycosylation of HA is a central regulator of influenza virus entry, replication, and immune evasion, mediated by specific glycosyltransferases. However, the enzyme responsible for N110 glycosylation in H3N2 HA remains unidentified. To address this, we performed co-immunoprecipitation (co-IP) coupled with MS to identify HA-interacting glycosyltransferases. B4GAT1 was exclusively detected in NSS HA complexes but not in YSN HA complexes, suggesting a potential role in N110 glycosylation (Figure 3A and Table S5). Notably, B4GAT1 was the only glycosyltransferase detected in our initial IP-MS analysis. This unique identification likely reflects its role as a primary adapter or scaffold that directly binds the HA-NSS motif with high affinity and stability under our stringent purification conditions, whereas other enzymatic partners may engage in transient or lower-abundance interactions that fell below the detection threshold of mass spectrometry. However, it has been reported that B4GAT1 does not directly regulate N-glycosylation. Lee PL et al. reported that B4GAT1 (also known as B3GNT1) can interact with B4GALT1 to cooperatively regulate N-glycosylation modifications [19,20,21]. Our validation revealed that among B4GALT family members, only B4GALT1 interacts with B4GAT1, and they co-localize within cells (Figure 3B,C). Therefore, we hypothesized that B4GAT1 and B4GALT1 may bind to HA and form a complex to modulate HA glycosylation. To validate this interaction, we conducted co-IP assays in HEK293T cells and confirmed that HA (YSN and NSS) forms a specific complex with B4GAT1 and B4GALT1, with significantly stronger affinity for NSS HA (Figure 3D). Endogenous co-IP experiments in A549 cells infected with YSN or NSS viruses further confirmed the preferential binding of B4GAT1 and B4GALT1 to NSS HA (Figure 3E). Further investigation showed that knockdown of B4GAT1 and B4GALT1 in A549 cells abolished the increase in HA molecular weight caused by N110 glycosylation (Figure 3F). Interestingly, the molecular weight of HA in knockdown cells was slightly lower than that of YSN HA in wild-type cells, suggesting that while the B4GAT1-B4GALT1 complex is crucial for the N110 site, it may also contribute to the processing of other nascent glycosylation sites on HA, reflecting a broader regulatory role in viral glycoprotein maturation (Figure 3F).

Given that hypoxia enhances N110 glycosylation, we hypothesized that hypoxia may upregulate B4GAT1 and B4GALT1 expression. Indeed, hypoxic conditions (5% O_2_) increased B4GAT1 and B4GALT1 protein and mRNA levels in both HEK293T and A549 cells (Figure 3G). However, we observed a discordance between the mRNA and protein levels of HIF-1α and B4GAT1. Consistent with previous reports, HIF-1α is regulated predominantly at the post-transcriptional level. B4GAT1 exhibited robust transcriptional induction with only a modest increase in protein. The discrepancy for B4GAT1 likely stems from two factors. First, it reflects a temporal disconnect between transcription and translation; transcriptional induction often precedes protein accumulation, and our detection time point may have captured the peak of mRNA expression while the protein was still in the early phase of synthesis. Second, the modest protein increase relative to the robust mRNA rise suggests post-transcriptional buffering. This indicates that B4GAT1 expression may be restricted by translation efficiency or rapid protein turnover. Moreover, hypoxia strengthened the formation of HA-B4GAT1-B4GALT1 complex (Figure 3H), collectively indicating that B4GAT1 and B4GALT1 mediate N110 glycosylation and that hypoxia amplifies this process to promote viral replication.

### 3.4. The B4GAT1-B4GALT1 Complex Modulates the Hypoxia-Induced Promotion of H3N2 Viral Replication

**Figure 4:**
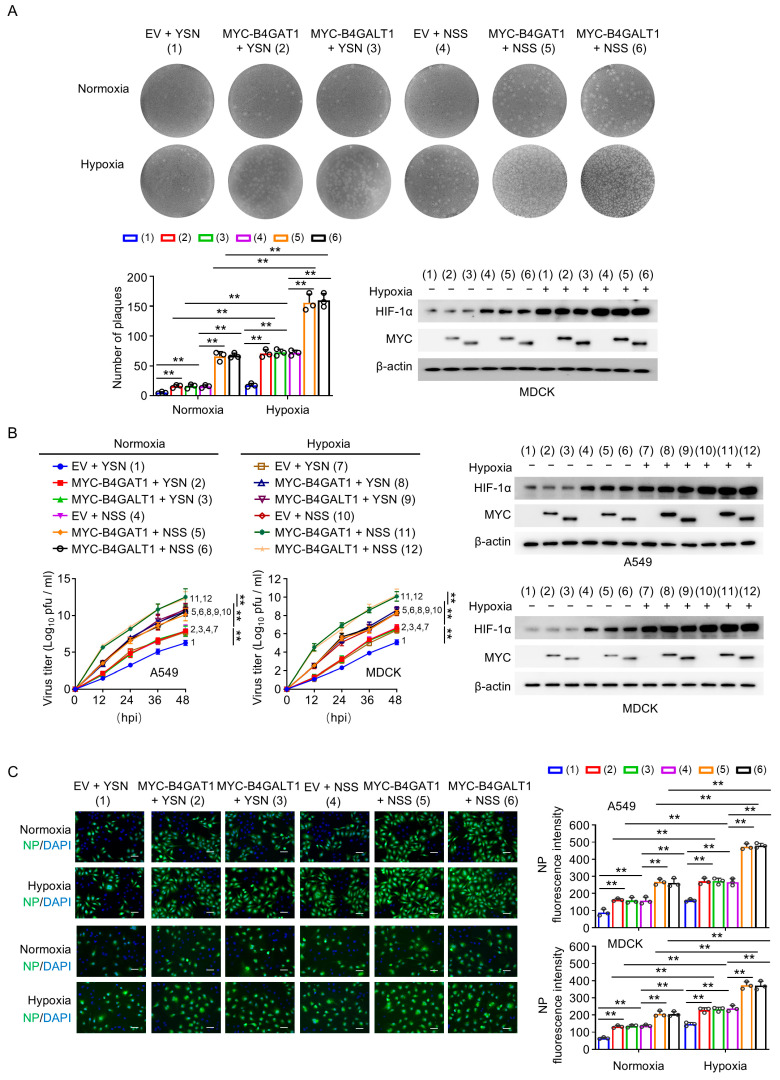
The B4GAT1-B4GALT1 Complex Modulates the Hypoxia-Induced Promotion of H3N2 Viral Replication. (**A**) MDCK cells were infected with virus at an MOI of 0.1 and transfected with plasmids as indicated under normoxia or hypoxia. Plaque formation was visualized by crystal violet staining (**Top**), and the number of plaques was quantified (**Bottom left**), circles indicate values from three replicates. Immunoblot analysis confirmed the expression of MYC-tagged enzymes and HIF-1α stabilization. β-actin served as a loading control (**Bottom right**). (**B**) Viral replication kinetics in A549 and MDCK cells. Cells were infected (MOI = 0.1) and transfected as described in (**A**). Supernatants were collected at indicated time points for titration. Immunoblot analysis shows the protein levels of the B4GAT1-B4GALT1 complex and HIF-1α in both cell lines. (**C**) Representative immunofluorescence images of viral NP protein (green) in infected A549 and MDCK cells (MOI = 3). Cells were transfected and maintained under normoxia or hypoxia, then fixed at 24 hpi. NP expression (green) was quantified by fluorescence intensity (**Right** panel), circles indicate values from three replicates. Nuclei were counterstained with DAPI (blue). Scale bar, 50 μm. Data are presented as mean ± SD of three independent experiments and were analyzed using one-way ANOVA with Bonferroni correction (**A**,**C**) and two-way repeated-measures ANOVA test with Bonferroni correction (**B**). ** *p* < 0.01.

To further elucidate the functional consequences of B4GAT1/B4GALT1-mediated N110 glycosylation, we evaluated their impact on viral fitness under both normoxic and hypoxic conditions. Plaque-forming assays in MDCK cells revealed that exogenous expression of B4GAT1 and B4GALT1 significantly increased the number and size of plaques for both YSN and NSS viruses (Figure 4A). Notably, while both variants benefited from the overexpression of these glycosyltransferases, the NSS virus exhibited a more pronounced increase in viral titers, consistent with its possession of the N110 glycosylation site.

We then monitored replication kinetics in A549 and MCDK cells. The results demonstrated that the B4GAT1-B4GALT1 complex substantially accelerated viral growth, leading to significantly higher titers at 12, 24, 36, and 48 h post-infection (hpi) compared to the control groups (Figure 4B). Crucially, the stimulatory effect of these enzymes was markedly amplified under hypoxia, which integrated with B4GAT1 and B4GALT1 to drive peak viral production (Figure 4B).

These findings were further corroborated by immunofluorescence (IF) staining targeting the viral nucleoprotein (NP). In both A549 and MDCK cell lines, the combined presence of hypoxia and B4GAT1/B4GALT1 overexpression resulted in a significantly higher percentage of NP-positive cells and increased fluorescence intensity (Figure 4C). Collectively, these data provide robust evidence that the B4GAT1-B4GALT1 complex is a critical host factor that promotes H3N2 replication, a process that is further exploited by the virus under hypoxic stress to maximize its infectivity.

### 3.5. N110 Glycosylation Modulates Pathogenicity and Cross-Protective Efficacy of H3N2 Viruses in Ferrets

**Figure 5:**
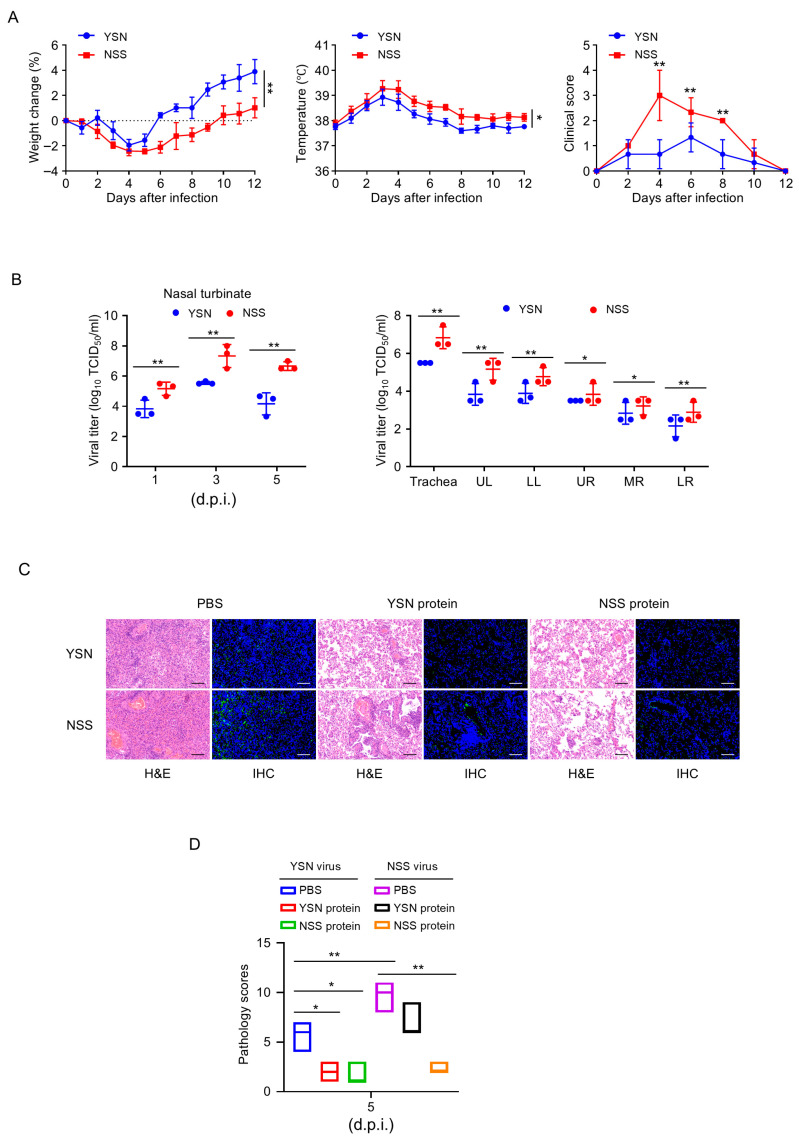
N110 glycosylation modulates pathogenicity and cross-protective efficacy of H3N2 viruses in ferrets. (**A**) Clinical parameters in ferrets intranasally infected with YSN or NSS viruses (*n* = 3). Body weight change, rectal temperature, and clinical scores were monitored daily for 12 days post-infection (dpi), the dashed line at the x-axis represents the baseline (zero value) (**Left** panel). (**B**) Viral titers in infected ferrets. **Left** panel: Viral titers in nasal swabs from YSN- or NSS-infected ferrets at 1, 3, and 5 dpi. **Right** panel: Viral titers in tracheal and lung tissues harvested at 5 dpi. UL, upper lobe. LL, lower lobe. UR, upper right lobe. MR, middle right lobe. LR, lower right lobe. (**C**) Hematoxylin-eosin (H&E) staining showing reduced inflammatory infiltration, alveolar damage, and interstitial edema in ferrets immunized with HA-NSS protein compared to HA-YSN or mock controls. Immunohistochemical staining (IHC) for viral NP (green) shows sparse NP^+^ cells in HA- or PBS-immunized lungs. Scale bar, 100 μm. (**D**) Pathology scores quantifying lung inflammation (alveolar damage, interstitial edema, inflammatory cell infiltration) and viral load, confirming superior protection by HA-NSS immunization. Data are presented as mean ± SD of three independent experiments and were analyzed using two-way repeated-measures ANOVA test with Bonferroni correction (**A**), Student’s *t* test (**B**) or one-way ANOVA test (**D**) with Bonferroni correction. * *p* < 0.05, ** *p* < 0.01.

To characterize viral virulence, ferrets were intranasally infected with YSN or NSS virus, and clinical parameters were monitored daily for 12 days. NSS-infected ferrets exhibited significantly greater weight loss, higher fever peaks, and elevated clinical scores compared to YSN-infected animals, with more severe respiratory symptoms (e.g., sneezing, labored breathing) observed in the NSS group (likely driven by N110 glycosylation-mediated viral fitness gains) (Figure 5A). Viral replication kinetics were assessed in the vaccine challenge experiment. NSS titers in nasal turbinates were significantly higher than YSN at 1, 3, and 5 days post-infection (dpi), with analogous trends in tracheal and lung tissues at 5 dpi (Figure 5B).

For protective efficacy evaluation, ferrets were immunized with recombinant HA-YSN or HA-NSS proteins prior to homologous viral challenge. At 5 dpi, lung histopathology revealed reduced inflammatory infiltration, alveolar damage, and interstitial edema in HA-immunized groups versus mock controls, with HA-NSS conferring superior protection against NSS challenge (Figure 5C). Immunohistochemical staining for viral nucleoprotein (NP) showed sparse NP^+^ cells in HA-immunized lungs versus dense NP expression in controls. Notably, regions with more severe inflammation correlated with higher numbers of NP^+^ cells, indicating increased viral load (Figure 5C). Pathology scores confirmed lower inflammation in HA-immunized ferrets. Importantly, HA-NSS immunization conferred robust cross-protection against both viruses, whereas HA-YSN showed slightly reduced efficacy against NSS virus challenge, highlighting the critical role of N110 glycosylation in shaping protective immune responses (Figure 5D).

### 3.6. N110 Glycosylation Regulates Cross-Reactive Antibody Responses and Neutralizing Efficacy of Post-Infection Sera

**Figure 6:**
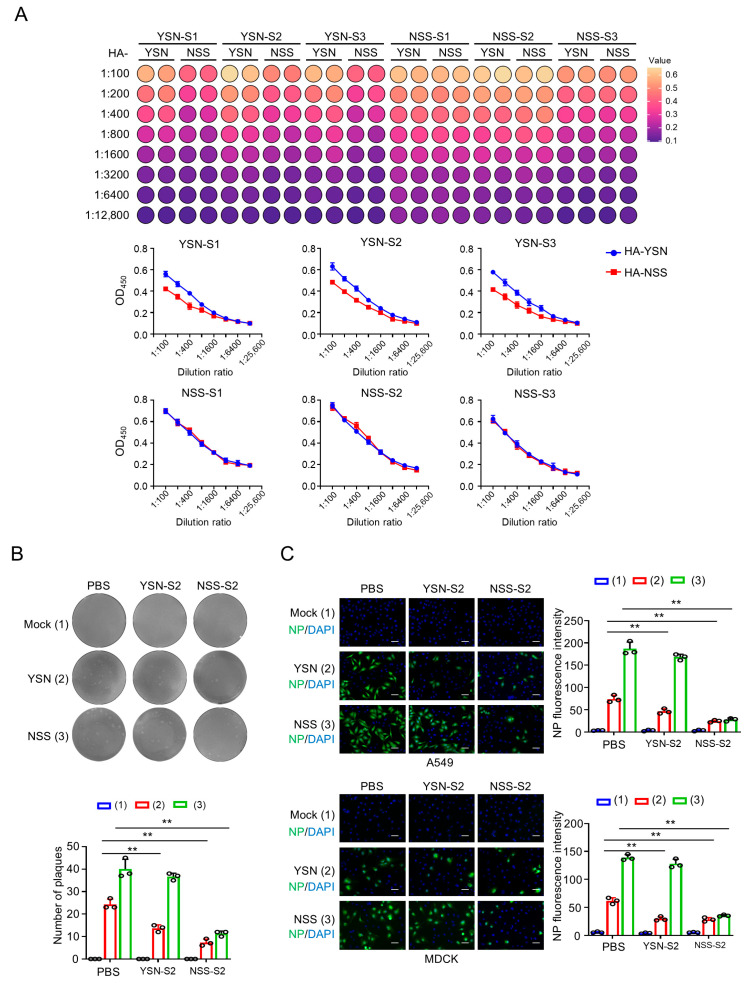
N110 glycosylation regulates cross-reactive antibody responses and neutralizing efficacy. (**A**) ELISA analysis of antibody binding in ferret sera. Sera were collected from ferrets infected with YSN or NSS virus (individual ferrets denoted as S1, S2, S3). The top heatmap visually represents the data in a color-coded format, and the bottom statistical chart quantitatively illustrates the comparison of serum reactivity against recombinant HA-YSN and HA-NSS proteins, as tested in serial dilutions. (**B**) MDCK cells were infected with virus at an MOI of 0.1 and treated with YSN-S2 or NSS-S2 as shown in (**A**). Plaque formation was visualized through crystal violet staining and the quantitative analysis of plaque number was determined using Image J (**Bottom** panel), circles indicate values from three replicates. (**C**) Representative images of immunofluorescence staining with the anti-NP antibody (green). A549 and MDCK cells were infected with virus at an MOI of 3 and treated with YSN-S2 or NSS-S2 as shown in (**A**). Then cells were fixed at 24 hpi for immunofluorescence staining. Nuclei were stained with DAPI (blue) and the quantitative analysis of NP protein fluorescence intensity was carried out (**Right** panel), circles indicate values from three replicates. Scale bar, 50 μm. Data are presented as mean ± SD of three independent experiments, and were analyzed using one-way ANOVA test (**B**,**C**) with Bonferroni correction. ** *p* < 0.01.

Given the N110 glycosylation-mediated high transmissibility and pathogenicity of NSS virus, we next evaluated whether serum antibodies induced by H3N2 virus infection in ferrets could confer protective immunity. Sera were collected from ferrets infected with YSN or NSS virus (YSN-S1-S3 and NSS-S1-S3). ELISA analysis revealed that YSN-infected sera bound both HA-YSN and HA-NSS proteins, with significantly stronger affinity for homologous HA-YSN (Figure 6A). In contrast, NSS-infected sera exhibited comparable binding to both HA proteins, with overall higher titers than YSN sera (Figure 6A). Based on these results, the highest-titer sera (YSN-S2 and NSS-S2) were selected for functional assays.

Plaque-forming assays in MDCK cells demonstrated that NSS-S2 sera potently inhibited plaque formation by both YSN and NSS viruses, whereas YSN-S2 sera showed markedly reduced efficacy against NSS virus (Figure 6B). Immunofluorescence assays in MDCK and A549 cells confirmed these findings, with NSS-S2 sera significantly reducing viral infection efficiency across cell types, while YSN-S2 sera failed to effectively neutralize NSS virus (Figure 6C). These data indicate that NSS infection elicits broadly reactive antibodies with cross-neutralizing activity against heterologous H3N2 viruses, whereas YSN-induced antibodies exhibit limited cross-protection, likely due to differential antigenic exposure driven by the N110 glycosylation motif in NSS HA. Thus, targeted vaccine design addressing the N110 glycosylation modification is critical for effectively inhibiting the infection and transmission of recently circulating H3N2 viruses.

## 4. Discussion

The glycosylation sites of influenza viruses, particularly those on the HA protein of H3N2 subtypes, exhibit a dynamic evolutionary pattern closely linked to viral immune evasion and adaptation. For instance, H5N6 influenza viruses possess an N-glycosylation site at position 158 of the HA protein, which promotes viral assembly and replication in various cell types while enhancing pathogenicity in mice, although its deletion increases thermal stability and viral transmission [22]. H1N1 viruses have shown site-specific glycosylation variations in HA, with increased glycan complexity in the globular head region, and NA protein glycosylation at position N73 with predominantly biantennary complex-type structures [23]. H5 and H9 subtypes demonstrate a higher potential to acquire new glycosylation sites through nucleotide mutations, suggesting greater adaptability to immune pressure compared to H7 subtypes [24]. In contrast, the HA protein of H3N2 influenza viruses has undergone significant glycosylation site expansion, primarily in the globular head region, driven by positive selection to shield antigenic epitopes and evade immune responses [25]. Early H3N2 strains contained only two N-glycosylation sites in the HA head domain, but subsequent evolution led to the accumulation of additional sites, including N144, which emerged as an immune escape mutation reducing neutralization sensitivity to human sera [10], and N158, whose glycosylation via the NYT motif blocks receptor binding and antibody recognition [8]. Further modifications include N142 and N174, identified in recent strains, which alter epitope structure and antibody affinity [26], while conserved sites like N165 and N285 with high-mannose glycans enhance interactions with surfactant protein D (SP-D) to promote viral clearance and reduce virulence [9]. Notably, the N110 glycosylation site identified in this study represents a functional departure from the previously characterized mechanisms. The structural modeling reveals that the N110 glycan is localized to a distal lateral surface approximately 32.1 Å from the RBS. Our experimental evidence demonstrates that rather than imposing steric hindrance, N110 glycosylation significantly enhances receptor-binding signal. This suggests a novel role for N110 as a non-shielding structural stabilizer that may optimize the HA head conformation or local biophysical environment to increase viral fitness. This finding highlights an evolutionary shift in H3N2 adaptation, where glycosylation serves not only as a passive shield for immune escape but also as an active modulator to refine receptor engagement. Continued surveillance of such non-canonical sites is essential to inform vaccine development and therapeutic strategies.

Hypoxia, primarily through the stabilization of HIF-1α, exerts multifaceted and virus-specific effects on viral invasion and host cell infection. For example, hypoxia reduces SARS-CoV-2 invasion by modulating ACE2 and ADAM17 via HIF-1α [27,28], promotes HBV persistence through A3B suppression and enhances replication via BCP transactivation [29,30], and boosts oncolytic herpesvirus replication by upregulating metabolic enzymes [31]. Beyond direct viral modulation, hypoxia also influences host protein glycosylation to alter cellular functions: it induces GLT8D1 to stabilize CD133 via N-glycosylation in glioma [32], suppresses FUT1/FUT2 to reduce fucosylation in pancreatic cancer [33], modifies PFK1 O-GlcNAcylation to regulate glycolysis [34], and upregulates glycosyltransferases to promote tumor migration [35]. However, whether hypoxia affects viral invasion and infection by altering viral protein glycosylation remains unreported. Our study is the first to demonstrate that hypoxia significantly enhances the N-linked glycosylation of the H3N2 HA protein, particularly by increasing the abundance of hybrid-type N-glycans at the N110 site. This modification enhances viral binding signal to host cell receptors and promotes efficient infection and replication in in vitro models. Furthermore, viral infections can actively modulate host hypoxic microenvironments to dysregulate cellular functions: H1N1 influenza virus stabilizes HIF-1α by inhibiting proteasomal degradation and reducing FIH-1 expression under normoxia [12], induces HIF-1α nuclear translocation to promote proinflammatory cytokine secretion [36], and upregulates HIF-1α/HK2-mediated glycolysis to enhance viral replication [13]. Respiratory viruses like SARS-CoV-2 also exacerbate hypoxic stress through inflammatory-HIF-1α crosstalk [37]. While our study focused on hypoxia-driven viral glycosylation (H3N2 HA) rather than viral modulation of host hypoxia, we observed that H3N2 infection significantly enhanced HIF-1α stability, with the NSS virus further augmenting this effect. However, the underlying mechanisms remain unclear. Future research should focus on these mechanisms and how viral–host hypoxic crosstalk impacts glycosylation-dependent viral fitness.

Glycosyltransferases play pivotal roles in regulating viral infection, protein folding, and immune evasion through viral protein glycosylation [38,39]. Oligosaccharyl transferase (OST) mediates the initial transfer of core glycans to asparagine residues, with mammalian OST isoforms STT3A and STT3B governing co-translational and post-translational N-linked glycosylation, respectively [40]. Similarly, mammalian homologs POMT1 and POMT2 must form heterodimers to facilitate essential O-mannosylation [41]. In our study, mass spectrometry and biochemical validation identified B4GAT1 as a critical binding partner of HA. While B4GAT1 is traditionally known as a β-1,4-glucuronyltransferase involved in α-dystroglycan functional glycosylation [20,42], recent evidence suggests it can interact with β-1,4-galactosyltransferase 1 (B4GALT1) to cooperatively regulate N-glycosylation [19]. Indeed, we demonstrated that B4GAT1 and B4GALT1 form a stable functional complex that mediates the N110 glycosylation of H3N2 HA. Interestingly, our results showed that knockdown of either B4GAT1 or B4GALT1 not only abolished the N110-linked glycan but also led to a reduction in the overall molecular weight of HA below its baseline levels, suggesting that this complex plays a broader regulatory role in the general N-glycosylation maturation of HA by processing multiple nascent sites. Nevertheless, the preferential recruitment of this enzymatic axis to the N110 site is driven by the significantly higher binding affinity of the HA-NSS variant for the B4GAT1-B4GALT1 complex compared to the YSN variant. Notably, hypoxia significantly upregulated both B4GAT1 and B4GALT1 at the mRNA and protein levels, while simultaneously strengthening the formation of the HA-B4GAT1-B4GALT1 ternary complex. This hypoxia-augmented interaction selectively intensified its impact on the HA-NSS variant, leading to superior replicative capacity and infectivity. Critically, the glycosylation changes mediated by this complex, particularly at sites like N110, may represent adaptations that are not only selected by immune pressure but also initiated by host cellular environments, such as hypoxia. These findings reveal a novel mechanism where the host microenvironment modulates viral fitness by inducing a specific glycosyltransferase complex, identifying the B4GAT1-B4GALT1 axis as a potential therapeutic target for intervening in the glycosylation-dependent viral life cycle. In our study, cellular hypoxia is confirmed to be a major and direct driver of glycosylation changes at the N110 site of the HA protein in H3N2 influenza virus. This perspective does not negate the well-established theory of immune-driven evolution but rather provides an important complement to it. First, immune selection pressure alone cannot adequately explain the observed changes in glycosylation intensity at the N110 site under hypoxic conditions. Second, growing evidence indicates that influenza virus infection actively induces a “pseudo-hypoxic” state. Specifically, studies have shown that the virus stabilizes the host transcription factor HIF-1α [12]. This stabilization triggers a cascade of events, including metabolic reprogramming toward glycolysis and lactate production, which in turn disrupts key innate immune signaling pathways (e.g., MAVS/RIG-I) to suppress interferon production [13]. Thus, hypoxia is not a passive consequence of inflammation but an active viral strategy that both promotes viral replication and suppresses innate immunity. Our study reveals that the localized hypoxic microenvironment induced by viral infection rapidly upregulates intracellular HIF-1α protein levels and enhances the expression of the glycosyltransferases B4GAT1 and B4GALT1. This mechanism acts directly on the host glycosylation machinery, operating independently of adaptive immune responses. Therefore, we propose a co-evolution model in which hypoxia-driven glycosylation may generate initial glycan diversity and form a protective shield, upon which immune selection subsequently acts to refine antigenicity. For H3N2 influenza virus, hypoxia is far from a minor factor—it is a crucial upstream physiological trigger that profoundly shapes the glycosylation landscape of HA, playing a significant role in fully understanding the evolutionary dynamics and adaptive advantages of the virus. It should be noted that certain methodological limitations exist in our study. In the receptor-binding ELISA, the binding curves did not reach full saturation (B_max_) under the tested concentrations, preventing the determination of precise affinity constants. Therefore, these results represent the apparent binding response under specific experimental conditions rather than absolute biophysical affinity. Future studies utilizing Surface Plasmon Resonance (SPR) or Bio-Layer Interferometry (BLI) will be required to further elucidate the kinetic parameters of these interactions. Additionally, while the combination of hypoxia and the B4GAT1-B4GALT1 Complex showed a markedly increased effect on viral replication, this was not formally tested for mathematical synergy. Thus, these observations should be interpreted as a collective enhancement rather than a formal synergistic interaction.

Viral protein glycosylation plays a pivotal role in influenza vaccine design by modulating antigenicity, immune evasion, and vaccine efficacy. Accumulation of N-linked glycans on the HA head domain, driven by immune selection, shields antigenic sites and enables antigenic drift, reducing vaccine effectiveness against rapidly evolving strains like H3N2 [43,44,45]. For example, egg-based vaccine production often induces HA mutations (e.g., loss of glycosylation site N160 in H3N2), leading to antigenic mismatch with circulating viruses and diminished neutralizing antibody responses [46,47]. Novel strategies such as “monoglycosylated HA” (HAmg) technology, which trims glycans to expose conserved epitopes, have shown promise in inducing broader cross-reactive immunity [48]. Glycosylation also influences viral subpopulation dynamics, as altered glycan profiles may affect particle assembly and infectivity, impacting live attenuated vaccine safety and efficacy [49]. In our study, sera from ferrets immunized with HA-NSS protein potently inhibited infection and replication of both YSN and NSS viruses, whereas sera from HA-YSN-immunized ferrets showed poor neutralization against NSS virus. This highlights the critical role of N110 glycosylation in shaping protective immune responses and vaccine design. Notably, the WHO-recommended quadrivalent influenza vaccines for 2024 and 2025 incorporate two H3N2 strains (A/Thailand/8/2022 and A/Croatia/10136RV/2023) that possess the NSS genotype and the associated N110 glycosylation site. Our research provides a theoretical basis for the effectiveness of this vaccine, reinforcing that timely vaccination with annually updated influenza vaccines is crucial to resisting infection, inhibiting transmission, and reducing seasonal influenza incidence.

## Data Availability

The data that support the findings of this study are available from the corresponding author upon request.

## Supplementary Materials

The following supporting information can be downloaded at: https://www.mdpi.com/article/10.3390/v18050547/s1, Table S1: Primers for reverse genetics plasmids; Table S2: Primers for expression vectors; Table S3: Primers for real-time PCR; Table S4: The sequences of shRNAs; Table S5: Proteins identified by mass spectrometry.
