# Supplementary material for: Hypoxia-Enhanced N110 Glycosylation of Hemagglutinin Promotes H3N2 Influenza Virus Fitness by Modulating Receptor Binding and Immune Evasion
Source: Viruses. 2026 May 8;18(5):547. doi: 10.3390/v18050547 (PMC13211402; doi:10.3390/v18050547)
Supplement: Supplementary file 1 [file viruses-18-00547-s001.zip › viruses-4274310-supplementary.pdf]

viruses

MDPI

Article

Hypoxia-Enhanced N110 Glycosylation of Hemagglutinin Promotes H3N2 Influenza Virus Fitness by Modulating Receptor Binding and Immune Evasion

Ting Zhang [1], Yihui Fang[1,2], Jie Liu[1], Ao Guo[3], Bin Yuan[3], Yanan Zhang [1,*], Lihua Ding [1,*] and Qinong Ye [1,*]

1 Academy of Military Medical Sciences, Beijing 100850, China
2 Department of Gastroenterology, General Hospital of Northern Theater Command, Shenyang 110840, China
3 Department of Pharmacology, School of Basic Medical Sciences, Anhui Medical University, Hefei, Anhui 230032, China
* Correspondence: zhangyn07@126.com (Y.Z.), dinglh2004@126.com (L.D.) and yeqn88@163.com (Q.Y.)

Supplementary Tables

Table S1. Primers for reverse genetics plasmids [14]

Gene	Forward (5′→3′)	Reverse (5′→3′)
PB2	AAGTTGGGGGGGagcgaaagcaggTC	GGTTATTagtagaaacaaggTCGTTT
PB1	AAGTTGGGGGGGagcgaaagcaggCA	GGTTATTagtagaaacaaggCATTT
PA	AAGTTGGGGGGGagcgaaagcaggTAC	GGTTATTagtagaaacaaggTACTT
NA	AAGTTGGGGGGGagcaaaagcaggAGT	GGTTATTagtagaaacaaggAGTTTTTT
HA	AAGTTGGGGGGGagcaaaagcaggGG	GGTTATTagtagaaacaaggGTGTTTT
NP	AAGTTGGGGGGGagcaaaagcaggGTA	GGTTATTagtagaaacaaggGTATTTTT
NS	AAGTTGGGGGGGagcaaaagcaggGTG	GGTTATTagtagaaacaaggGTGTTTT
M	AAGTTGGGGGGGagcaaaagcaggTAG	GGTTATTagtagaaacaaggTAGTTTT
CPEC (G)	CGAAGTTGGGGGGGagcgaaagcagg	
CPEC (A)	CGAAGTTGGGGGGGagcaaaagcagg	

 https://doi.org/10.3390/v18050547

Table S2. Primers for expression vectors

Gene	Species	Forward (5′→3′)	Reverse (5′→3′)
MYC-B4GAT1	Human	TTGCGGAAACATATGGGATCCGCCACC ATGCAGATGTCCTACGCCAT	GCCCGGGGCGGCCGCCTCGAG GCAGCGTCGGGGAGAGTT
FLAG-B4GAT1	Human	TTGGTACCGAGCTCGGATCCGCCACC ATGCAGATGTCCTACGCCAT	AAGGGCCCTCTAGACTCGAG GCAGCGTCGGGGAGAGTT
V5-B4GALT1	Human	TTGGTACCGAGCTCGGATCCGCCACC ATGAGGCTTCGGGAGCCG	GGGATAGGCTTACCCTCGAG GCTCGGTGTCCCGATGTC
MYC-B4GALT1	Human	TTGCGGAAACATATGGGATCCGCCACC ATGAGGCTTCGGGAGCCG	GCCCGGGGCGGCCGCCTCGAG GCTCGGTGTCCCGATGTC
MYC-B4GALT2	Human	TTGCGGAAACATATGGGATCCGCCACC ATGGCTGTGGAAGTCCAGGA	GCCCGGGGCGGCCGCCTCGAG GCCCCGAGGGGGCCACGA
MYC-B4GALT3	Human	TTGCGGAAACATATGGGATCCGCCACC ATGTTGCGGAGGCTGCTG	GCCCGGGGCGGCCGCCTCGAG GTGTGAAC-CTCGGAGGGC
MYC-B4GALT5	Human	TTGCGGAAACATATGGGATCCGCCACC ATGCGCGCCCGCCGGGGG	GCCCGGGGCGGCCGCCTCGAG GTACTCGTTCACCTGAGCCAG
FLAG-HA-YSN	Human	TTGGTACCGAGCTCGGATCCGCCACC ATGAAGACCATCATTGCTTTG	AAGGGCCCTCTAGACTCGAG AATGCAAATGTTGCATCTAATG
HA-NSS variant/ FLAG-HA-NSS	Human	AGCCaacagcagcTGTTA CCCTTATGATGTGCCGG	AACAgctgctgttGGCTC TGCTTCGTTCAACAAA

Table S3. Primers for real-time PCR

Gene	Species	Forward (5′→3′)	Reverse (5′→3′)
B4GAT1	Human	CAGTACGGCTTCAACCGAAT	ACTTGGCCTTCAACTCCTGT
B4GALT1	Human	AGGTGTCTCTGCTCTAAGT	TCCCGACCACAGCATTTG
HIF-1α	Human	ATCCATGTGACCATGAGGAAATG	TCGGCTAGTTAGGGTACACTTC
β-actin	Human	TCGTGCGTGACATTAAGGAG	ATGCCAGGGTACATGGTGGT

https://doi.org/10.3390/v18050547

Table S4. The sequences of shRNAs

Gene	Species	Sequence (5′→3′)
B4GAT1	Human	GTTTCTTGGTTCATAAGGGCT
B4GALT1	Human	GATACCCATTGTATACCCAAA

https://doi.org/10.3390/v18050547

Table S5. Proteins identified by mass spectrometry

Accession	Gene Name	Protein descriptioon	YSN	NSS
O15020	SPTBN2	Spectrin beta chain, non-erythrocytic 2	1	0
O15371	EIF3D	Eukaryotic translation initiation factor 3 subunit D	1	0
O43242	PSMD3	26S proteasome non-ATPase regulatory subunit 3	1	0
O60313	OPA1	Dynamin-like GTPase OPA1, mitochondrial	1	0
O75821	EIF3G	Eukaryotic translation initiation factor 3 subunit G	1	0
O75934	BCAS2	Pre-mRNA-splicing factor SPF27	1	0
P04908	H2AC8	Histone H2A type 1-B/E	1	0
P07477	PRSS1	Serine protease 1	1	0
P08237	PFKM	ATP-dependent 6-phosphofructokinase, muscle type	1	0
P12110	COL6A2	Collagen alpha-2(VI) chain	1	0
P24534	EEF1B2	Elongation factor 1-beta	1	0
P35580	MYH10	Myosin-10	1	0
P35606	COPB2	Coatomer subunit beta'	1	0
P39687	ANP32A	Acidic leucine-rich nuclear phosphoprotein 32 family member A	1	0
P55884	EIF3B	Eukaryotic translation initiation factor 3 subunit B	1	0
P84085	ARF5	ADP-ribosylation factor 5	1	0
P85037	FOXK1	Forkhead box protein K1	1	0
Q01650	SLC7A5	Large neutral amino acids transporter small subunit 1	1	0
Q14315	FLNC	Filamin-C	1	0
Q14739	LBR	Delta(14)-sterol reductase LBR	1	0
Q16658	FSCN1	Fascin	1	0
Q8NF37	LPCAT1	Lysophosphatidylcholine acyltransferase 1	1	0
Q8TEX9	IPO4	Importin-4	1	0
Q8WWC4	MAIP1	m-AAA protease-interacting protein 1, mitochondrial	1	0
Q96P70	IPO9	Importin-9	1	0
Q9C037	TRIM4	E3 ubiquitin-protein ligase TRIM4	1	0
Q9H0S4	DDX47	Probable ATP-dependent RNA helicase DDX47	1	0
Q9NQ55	PPAN	Suppressor of SWI4 1 homolog	1	0
Q9P2E9	RRBP1	Ribosome-binding protein 1	1	0
Q9P2N7	KLHL13	Kelch-like protein 13	1	0
Q9UBX3	SLC25A10	Mitochondrial dicarboxylate carrier	1	0
O00411	POLRMT	DNA-directed RNA polymerase, mitochondrial	0	1
O15541	RNF113A	E3 ubiquitin-protein ligase RNF113A	0	1
O43505	B4GAT1	Beta-1,4-glucuronyltransferase 1	0	1
P12109	COL6A1	Collagen alpha-1(VI) chain	0	1
P13473	LAMP2	Lysosome-associated membrane glycoprotein 2	0	1
P17480	UBTF	Nucleolar transcription factor 1	0	1
P21291	CSRP1	Cysteine and glycine-rich protein 1	0	1
P27695	APEX1	DNA repair nuclease/redox regulator APEX1	0	1
P30048	PRDX3	Thioredoxin-dependent peroxide reductase, mitochondrial	0	1
P35590	TIE1	Tyrosine-protein kinase receptor Tie-1	0	1
P36957	DLST	Dihydrolipoyllysine-residue succinyltransferase component of 2-oxoglutarate dehydrogenase complex, mitochondrial	0	1
P37837	TALDO1	Transaldolase	0	1
P55854	SUMO3	Small ubiquitin-related modifier 3	0	1
P62854	RPS26	Small ribosomal subunit protein eS26	0	1
Q01130	SRSF2	Serine/arginine-rich splicing factor 2	0	1
Q08945	SSRP1	FACT complex subunit SSRP1	0	1
Q09028	RBBP4	Histone-binding protein RBBP4	0	1
Q15772	SPEG	Striated muscle preferentially expressed protein kinase	0	1
Q16555	DPYSL2	Dihydropyrimidinase-related protein 2	0	1

https://doi.org/10.3390/v18050547

Q5JPH6	EARS2	Nondiscriminating glutamyl-tRNA synthetase EARS2, mitochondrial	0	1
Q969V3	NCLN	BOS complex subunit NCLN	0	1
Q96HU8	DIRAS2	GTP-binding protein Di-Ras2	0	1
Q9BUF5	TUBB6	Tubulin beta-6 chain	0	1
Q9H5Q4	TFB2M	Dimethyladenosine transferase 2, mitochondrial	0	1
Q9NNW5	WDR6	tRNA (34-2'-O)-methyltransferase regulator WDR6	0	1
Q9NSB2	KRT84	Keratin, type II cuticular Hb4	0	1
Q9NYF8	BCLAF1	Bcl-2-associated transcription factor 1	0	1
Q9UBS4	DNAJB11	DnaJ homolog subfamily B member 11	0	1
Q9Y2T2	AP3M1	AP-3 complex subunit mu-1	0	1
Q9Y5F7	PCDHGC4	Protocadherin gamma-C4	0	1
Q96IV0	NGLY1	Peptide-N(4)-(N-acetyl-beta-glucosaminyl) asparagine amidase	1	1
A0A075B6R9	IGKV2D-24	Probable non-functional immunoglobulin kappa variable 2D-24	1	1
Q9NYU2	UGGT1	UDP-glucose:glycoprotein glucosyltransferase 1	1	1
A0A8I5KQE6	RPSA2	Small ribosomal subunit protein uS2B	1	1
A0FGR8	ESYT2	Extended synaptotagmin-2	1	1
O00165	HAX1	HCLS1-associated protein X-1	1	1
O14773	TPP1	Tripeptidyl-peptidase 1	1	1
O14776	TCERG1	Transcription elongation regulator 1	1	1
O43390	HNRNPR	Heterogeneous nuclear ribonucleoprotein R	1	1
O60830	TIMM17B	Mitochondrial import inner membrane translocase subunit Tim17-B	1	1
O95772	STARD3NL	STARD3 N-terminal-like protein	1	1
O96005	CLPTM1	Putative lipid scramblase CLPTM1	1	1
P01859	IGHG2	Immunoglobulin heavy constant gamma 2	1	1
P07951	TPM2	Tropomyosin beta chain	1	1
P11166	SLC2A1	Solute carrier family 2, facilitated glucose transporter member 1	1	1
P23381	WARS1	Tryptophan--tRNA ligase, cytoplasmic	1	1
P24666	ACP1	Low molecular weight phosphotyrosine protein phosphatase	1	1
P27824	CANX	Calnexin	1	1
P46020	PHKA1	Phosphorylase b kinase regulatory subunit alpha, skeletal muscle isoform	1	1
P49593	PPM1F	Protein phosphatase 1F	1	1
P53007	SLC25A1	Tricarboxylate transport protein, mitochondrial	1	1
P53985	SLC16A1	Monocarboxylate transporter 1	1	1
P57088	TMEM33	Transmembrane protein 33	1	1
Q07065	CKAP4	Cytoskeleton-associated protein 4	1	1
Q13257	MAD2L1	Mitotic spindle assembly checkpoint protein MAD2A	1	1
Q14103	HNRNPD	Heterogeneous nuclear ribonucleoprotein D0	1	1
Q14204	DYNC1H1	Cytoplasmic dynein 1 heavy chain 1	1	1
Q14974	KPNB1	Importin subunit beta-1	1	1
Q7KZF4	SND1	Staphylococcal nuclease domain-containing protein 1	1	1
Q8N1F7	NUP93	Nuclear pore complex protein Nup93	1	1
Q9BU76	MMTAG2	Multiple myeloma tumor-associated protein 2	1	1
Q9NXS2	QPCTL	Glutaminyl-peptide cyclotransferase-like protein	1	1
Q9Y597	KCTD3	BTB/POZ domain-containing protein KCTD3	1	1
Q9Y5L0	TNPO3	Transportin-3	1	1

0: undetected
1: detected
